# Supplementary material for: Host-derived organic acids enable gut colonization of the honey bee symbiont Snodgrassella alvi
Source: Nat Microbiol. 2024 Jan 15;9(2):477–89. doi: 10.1038/s41564-023-01572-y (PMC11343714; doi:10.1038/s41564-023-01572-y)
Supplement: Supplementary file 1 — Supplementary Fig. 1, Tables 1 and 2 and protocol. [file 41564_2023_1572_MOESM1_ESM.pdf]

nature microbiology

Article

https://doi.org/10.1038/s41564-023-01572-y

Host-derived organic acids enable gut colonization of the honey bee symbiont *Snodgrassella alvi*

In the format provided by the authors and unedited

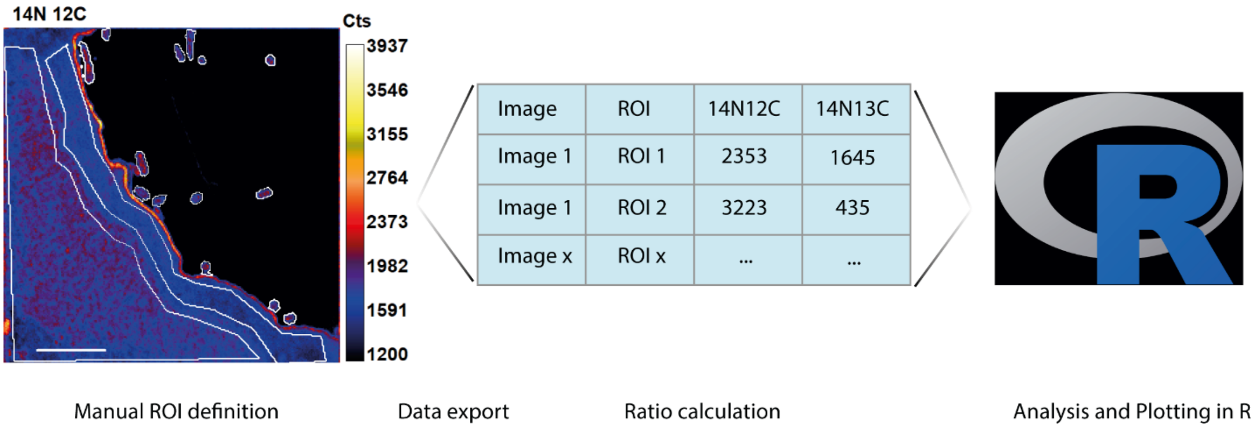

Supplementary Figure 1. NanoSIMS image analysis workflow. For each image, 10 layers were acquired by bombarding the surface of the samples with Cs+ Ion beam (See methods). The image processing was performed using "L'image" software (Larry Nittler, Carnegie Institution of Washington). The 10 layers were aligned and stacked and 44 ms deadtime correction was applied. Regions of interests (ROI) were manually drawn around bacterial cells, gut epithelium layer, and bulk host cells, using a minimum threshold specified and values were extracted and reported in **Supplementary table 3**. At% enrichment was calculated in R (See Code Availability). At% enrichment formula: $13C\ At\% = {}^{\text{14N 13C}}/_{(\text{14N 13C} + \text{14N 12C})} \times 100$.

	Replicate	1	2	3	4	5	Total
Bees	MD	34	24	24	24	0	106
	MD (P)	30	23	23	25	0	101
	Sn	31	24	20	0	20	95
	Sn (P)	34	23	20	0	19	96
	Sn+Gi	32	22	24	0	16	94
	Sn+Gi (P)	29	24	20	0	22	95
	Gi	0	0	0	26	25	51
	Gi (P)	0	0	0	25	25	50
Survival (%)	MD	100%	100%	100%	96%	N/A	99%
	MD (P)	100%	100%	100%	100%	N/A	100%
	Sn	94%	100%	100%	N/A	100%	98%
	Sn (P)	100%	100%	95%	N/A	100%	99%
	Sn+Gi	97%	95%	100%	N/A	100%	98%
	Sn+Gi (P)	97%	92%	95%	N/A	100%	96%
	Gi	N/A	N/A	N/A	100%	100%	100%
	Gi (P)	N/A	N/A	N/A	96%	100%	98%
CFU	MD	10	10	10	10	N/A	40
	MD (P)	10	10	10	10	N/A	40
	Sn	10	10	10	N/A	6	36
	Sn (P)	10	10	10	N/A	6	36
	Sn+Gi	10	10	10	N/A	6	36
	Sn+Gi (P)	10	10	10	N/A	6	36
	Gi	N/A	N/A	N/A	10	18	28
	Gi (P)	N/A	N/A	N/A	10	18	28
qPCR	MD	4	3	4	6	N/A	17
	MD (P)	4	3	4	5	N/A	16
	Sn	4	9	6	N/A	6	25
	Sn (P)	4	9	8	N/A	6	27
	Sn/Gi	10	10	11	N/A	6	37
	Sn/Gi (P)	10	10	10	N/A	6	36
	Gi	N/A	N/A	N/A	10	14	24
	Gi (P)	N/A	N/A	N/A	9	21	30
Metabolomics	MD	2	1	2	5	N/A	10
	MD (P)	2	1	4	5	N/A	12
	Sn	1	1	2	N/A	6	10
	Sn (P)	1	1	3	N/A	6	11
	Sn+Gi	3	1	5	N/A	6	15
	Sn+Gi (P)	1	1	4	N/A	5	11
	Gi	N/A	N/A	N/A	5	9	14
	Gi (P)	N/A	N/A	N/A	6	7	13
Pollen Consumption (mg/bee)	MD	-	-	-	-	-	-
	MD (P)	30	33	32	13	N/A	26
	Sn	-	-	-	-	-	-
	Sn (P)	20	31	30	N/A	22	26
	Sn+Gi	-	-	-	-	-	-
	Sn+Gi (P)	19	30	33	N/A	16	24
	Gi	-	-	-	-	-	-
	Gi (P)	N/A	N/A	N/A	17	18	17

Supplementary Table 1. *S. alvi* & *Gilliamella* colonization summary counts. Total bee numbers per experiment, survival rate, number of bees used for CFU plating, qPCR and metabolomics analysis, and pollen consumption are shown.

	Replicate	1	2	Total
Bees	MD	14	15	29
	wkB2	14	14	28
	251	16	15	31
	304	14	15	29
	323	14	15	29
	892	14	15	29
	897	15	14	29
Survival (%)	MD	93%	100%	96%
	wkB2	100%	100%	100%
	251	88%	100%	94%
	304	100%	80%	90%
	323	100%	100%	100%
	892	86%	100%	93%
	897	80%	79%	79%
CFU	MD	12	12	24
	wkB2	12	12	24
	251	11	12	23
	304	12	9	21
	323	12	12	24
	892	12	12	24
	897	12	9	21
Metabolomics	MD	6	6	12
	wkB2	6	6	12
	251	8	9	17
	304	6	6	12
	323	7	6	13
	892	6	6	12
	897	6	6	12

Supplementary Table 2. Summary of the number of bees sampled per replicate and treatment for the mono-colonization experiment with divergent *Snodgrassella* strains. Total number of bees per experiment, survival rate, and number of bees used for CFU plating and metabolomics analysis are shown.

Bee9: An M9 derived medium for bee gut bacteria

Description

This protocol is used to prepare stock solutions for an M9-based 'minimal' medium containing trace metals and vitamins, without casamino acids. Additional vitamins, and growth nutrients are added for growth of S. alvi and/or Gilliamella strains. The pH should be adjusted to 5.5 – 6.0

1. Preparation of HMB (hutner's mineral base) modified metals 44 (component 1)

- Weight on a balance the following:
  1. 1.095g $ZnSO_4$ x $7H_2O$, dissolve in **5ml** ddH_2O at pH=2
  2. 0.914g $FeSO_4$ x $7H_2O$, dissolve in **5ml** ddH_2O at pH=2
  3. 0.154g $MnSO_4$ x H_2O, dissolve in **5ml** ddH_2O at pH=2
  4. 0.392g $CuSO_4$ x $5H_2O$, dissolve in **100ml** ddH_2O at pH=2
  5. 0.248g $Co(NO_3)_2$ x $6H_2O$, dissolve in **100ml** ddH_2O at pH=2
  6. 0.177g $Na_2B_4O_7$ x $10H_2O$, dissolve in **100ml** ddH_2O at pH=2
- Mix **1.**, **2.**, **3.**, and add 10ml of each of **4.**, **5.**, **6**. to obtain **component 1**.
- Adjust volume to 100ml and sterile-filter with 0.22 um filter
- Keep it at 4C, light protected

2. Preparation of additional salts (components 2, 3, 4, 5)

- **Component 2:** 1.72g of $MgSO_4$ x $7H_2O$, dissolve in **100ml** ddH_2O at pH=2, sterile-filter with 0.22 um filter
- **Component 3:** 3.33g of $CaCl_2$ x $2H_2O$, dissolve in **100ml** ddH_2O at pH=2, sterile-filter with 0.22 um filter
- **Component 4:** 0.99g of $FeSO_4$ x $7H_2O$, dissolve in **100ml** ddH_2O at pH=2, sterile-filter with 0.22 um filter
- **Component 5:** 0.974g of $(NH_4)_6Mo_7O_{24}$ x $4H_2O$, dissolve in **100ml** ddH_2O at pH=2, sterile-filter with 0.22 um filter
- Keep at RT, light protected

3. Preparation of the vitamin 10x stock solution (component 6a)

- 0.2g of Calcium pantothenate, dissolve in **10ml** ddH_2O
- 0.01g of Thiamine-HCl, dissolve in **10ml** ddH_2O
- 0.01g of biotin, dissolve in **10ml** ddH_2O
- Mix 79ml of ddH_2O, 10ml of **2.**, 10ml of **3.**, and 1ml of **4.**
- Sterile-filter the final 100 ml of vitamin stock solution (component 6a)
- Component 6a can be kept for at least 6 months at 4°C in the fridge, protected from light

5. Preparation of 10x stock solution of M9 (component 8)

- Mix the following:
- 60g Na_2HPO_4

- 30g KH_2PO_4
- 5g NaCl
- 10g NH_4Cl
- Add water to 1l, autoclave
- Keep at RT

6. Preparation of *S. alvi* specific vitamins

- Weigh out powder of the following compounds and dissolve in M9 base to a concentration of 10 mM (for final concentration of [0.1 mM]
- 4-Hydroxybenzoate
- P-aminobenzoic acid
- Pyridoxine*HCl
- Choline

7. Prepare 500ml of M9 base medium

- Prepare the final M9 base medium freshly on the day of use
- For 500ml mix the following:
- Component 1: 500 µl
- Component 2: 8390 µl
- Component 3: 1000 µl
- Component 4: 100 µl
- Component 5: 10 µl (100 uL of 1/10 component 5 for small M9 volume)
- Component 6a (10x Vitamin stock): 50ml
- Component 8 (10x M9 stock): 50ml
- Custom vitamins (10mM stock) 5 mL
- ddH_2O: 385 ml

8. Prepare carbon + media

- Dissolve compounds of choice in M9 to a final concentration of 10 mM. Citrate is a good single carbon source. Glucose [10 mM] can also be added for *Gilliamella* growth.
- Common substrates include.
    - Alpha-ketoglutaric acid
    - Fumaric acid
    - Malic acid
    - Succinic acid
    - Citric acid
    - Sodium pyruvate
    - Sodium acetate
    - Sodium lactate
  - Check pH and adjust to 5.5 to 6.0
