# Supplementary material for: Host-derived organic acids enable gut colonization of the honey bee symbiont Snodgrassella alvi
Source: Nat Microbiol. 2024 Jan 15;9(2):477–89. doi: 10.1038/s41564-023-01572-y (PMC11343714; doi:10.1038/s41564-023-01572-y)
Supplement: Supplementary file 2 — Reporting Summary [file 41564_2023_1572_MOESM2_ESM.pdf]

nature portfolio

Corresponding author(s): Philipp Engel

Last updated by author(s): Nov 3, 2023

Reporting Summary

Nature Portfolio wishes to improve the reproducibility of the work that we publish. This form provides structure for consistency and transparency in reporting. For further information on Nature Portfolio policies, see our Editorial Policies and the Editorial Policy Checklist.

Statistics

For all statistical analyses, confirm that the following items are present in the figure legend, table legend, main text, or Methods section.

n/a	Confirmed	
☐	☒	The exact sample size (*n*) for each experimental group/condition, given as a discrete number and unit of measurement
☐	☒	A statement on whether measurements were taken from distinct samples or whether the same sample was measured repeatedly
☐	☒	The statistical test(s) used AND whether they are one- or two-sided<br>*Only common tests should be described solely by name; describe more complex techniques in the Methods section.*
☒	☐	A description of all covariates tested
☐	☒	A description of any assumptions or corrections, such as tests of normality and adjustment for multiple comparisons
☐	☒	A full description of the statistical parameters including central tendency (e.g. means) or other basic estimates (e.g. regression coefficient) AND variation (e.g. standard deviation) or associated estimates of uncertainty (e.g. confidence intervals)
☐	☒	For null hypothesis testing, the test statistic (e.g. *F*, *t*, *r*) with confidence intervals, effect sizes, degrees of freedom and *P* value noted<br>*Give P values as exact values whenever suitable.*
☒	☐	For Bayesian analysis, information on the choice of priors and Markov chain Monte Carlo settings
☒	☐	For hierarchical and complex designs, identification of the appropriate level for tests and full reporting of outcomes
☒	☐	Estimates of effect sizes (e.g. Cohen's *d*, Pearson's *r*), indicating how they were calculated

Our web collection on statistics for biologists contains articles on many of the points above.

Software and code

Policy information about availability of computer code

Data collection	Metabolomics: Masshunter Workstation Unknown Analysis software version 10.0 (Agilent) and the NIST 2017 MS library, MassHunter Workstation Quantitative Analysis software version 10.0 (Agilent).<br>NanoSIMS acquisition : Cameca Software NanoSIMS version 4.5
Data analysis	Metabolomics: R studio (4.0.3) including the lmm2met v1.0 package;<br>Phylogenetic tree : MUSCLE v3.8.1551 by Robert C. Edgar; IQ-TREE multicore version 2.0.3 for Linux 64-bit built Dec 20 2020 and the ITOL online viewer (https://itol.embl.de/)<br>NanoSIMS: “L’image” software version 10-15-2021 (Larry Nittler, Carnegie Institution of Washington)<br>Codes used to analyze the data and generate figures are available at:<br>https://doi.org/10.5281/zenodo.10066636

For manuscripts utilizing custom algorithms or software that are central to the research but not yet described in published literature, software must be made available to editors and reviewers. We strongly encourage code deposition in a community repository (e.g. GitHub). See the Nature Portfolio guidelines for submitting code & software for further information.

Data

Policy information about availability of data

All manuscripts must include a data availability statement. This statement should provide the following information, where applicable:

- Accession codes, unique identifiers, or web links for publicly available datasets
- A description of any restrictions on data availability
- For clinical datasets or third party data, please ensure that the statement adheres to our policy

The kynureninase protein sequence was retrieved from the publicly accessible NCBI database (WP 025330329.1). Raw data is available on zenodo at the following link: https://doi.org/10.5281/zenodo.10066636

Human research participants

Policy information about studies involving human research participants and Sex and Gender in Research.

Reporting on sex and gender	N/A
Population characteristics	N/A
Recruitment	N/A
Ethics oversight	N/A

Note that full information on the approval of the study protocol must also be provided in the manuscript.

Field-specific reporting

Please select the one below that is the best fit for your research. If you are not sure, read the appropriate sections before making your selection.

☒ Life sciences ☐ Behavioural & social sciences ☐ Ecological, evolutionary & environmental sciences

For a reference copy of the document with all sections, see nature.com/documents/nr-reporting-summary-flat.pdf

Life sciences study design

All studies must disclose on these points even when the disclosure is negative.

Sample size	Sample sizes differ throughout as detailed in the Material and Methods section. Sample sizes were determined based on availability/ opportunity, and are provided in each case as an exact sample size (n).
Data exclusions	Bacterial abundance quantification with qPCR: Samples with SD higher than 0.7 were excluded from the analysis<br>Metabolomics: Samples were removed from the metabolomics dataset with internal standards > or < 2*SD from the median of that batch. [Figure 1, n=6; Figure 5, n=3].
Replication	Colonization experiments were successfully replicated five time using bees from separate hives as described in the methods. In vitro experiments were replicated independently 3-4 times. Metabolomic analysis of MF bee guts and 13C enrichment analysis of MF bees fed 13C glucose were not replicated. Feeding of MF bees with 13C glucose, followed by colonization with S. alvi was not replicated, but a prior pilot experiment to establish the correct experimental timing showed similar results.
Randomization	For every bee experiment, bees were randomly assigned to cages.<br>Metabolomics and qPCR samples were randomly extracted and analyzed after the experiments were conducted
Blinding	No blinding was performed for in vivo experiments, as the dietary condition (+/-Pollen) could not be masked. In vitro conditions were not blinded, but handled simultaneously in batches.

Reporting for specific materials, systems and methods

We require information from authors about some types of materials, experimental systems and methods used in many studies. Here, indicate whether each material, system or method listed is relevant to your study. If you are not sure if a list item applies to your research, read the appropriate section before selecting a response.

Materials & experimental systems

n/a	Involved in the study
☒	☐ Antibodies
☒	☐ Eukaryotic cell lines
☒	☐ Palaeontology and archaeology
☐	☒ Animals and other organisms
☒	☐ Clinical data
☒	☐ Dual use research of concern

Methods

n/a	Involved in the study
☒	☐ ChIP-seq
☒	☐ Flow cytometry
☒	☐ MRI-based neuroimaging

Animals and other research organisms

Policy information about studies involving animals; ARRIVE guidelines recommended for reporting animal research, and Sex and Gender in Research

Laboratory animals	No laboratory animals were used in this study.
Wild animals	No wild animals were used in this study.
Reporting on sex	Newly emerged female workers bees were used. Results shown in the manuscript therefore apply only for female worker bees of the species Apis mellifera carnica.
Field-collected samples	Pupae of Apis mellifera carnica were removed from managed hives at the University of Lausanne. Pupae and emerged adult bees were reared in cages in temperature (33 C) and humidity (70% Rel.) controlled incubators and provided sucrose solution ad libitum. Prior to dissection, bees were anesthetized with CO2 and placed on ice. Wild bumble bees of species Bombus terrestris were caught on the campus of the university of Lausanne, sacrificed in the same manner as A. mellifera, and used to isolate bacterial strains.
Ethics oversight	No ethical approval was required for this study.

Note that full information on the approval of the study protocol must also be provided in the manuscript.
