# Supplementary figures and images for: Host-derived organic acids enable gut colonization of the honey bee symbiont Snodgrassella alvi
Source: Nat Microbiol. 2024 Jan 15;9(2):477–89. doi: 10.1038/s41564-023-01572-y (PMC11343714; doi:10.1038/s41564-023-01572-y)

24h 13C

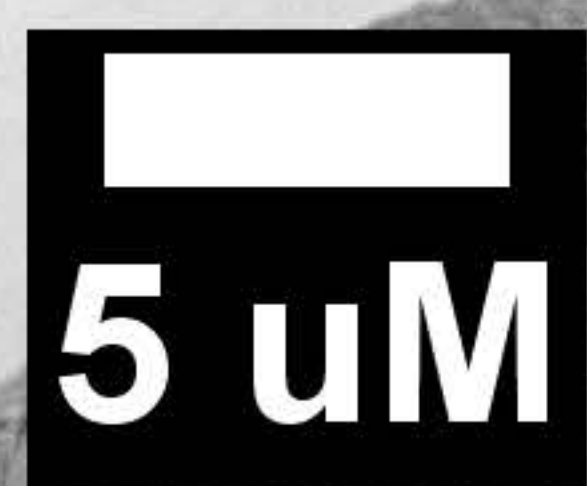

32h 13C

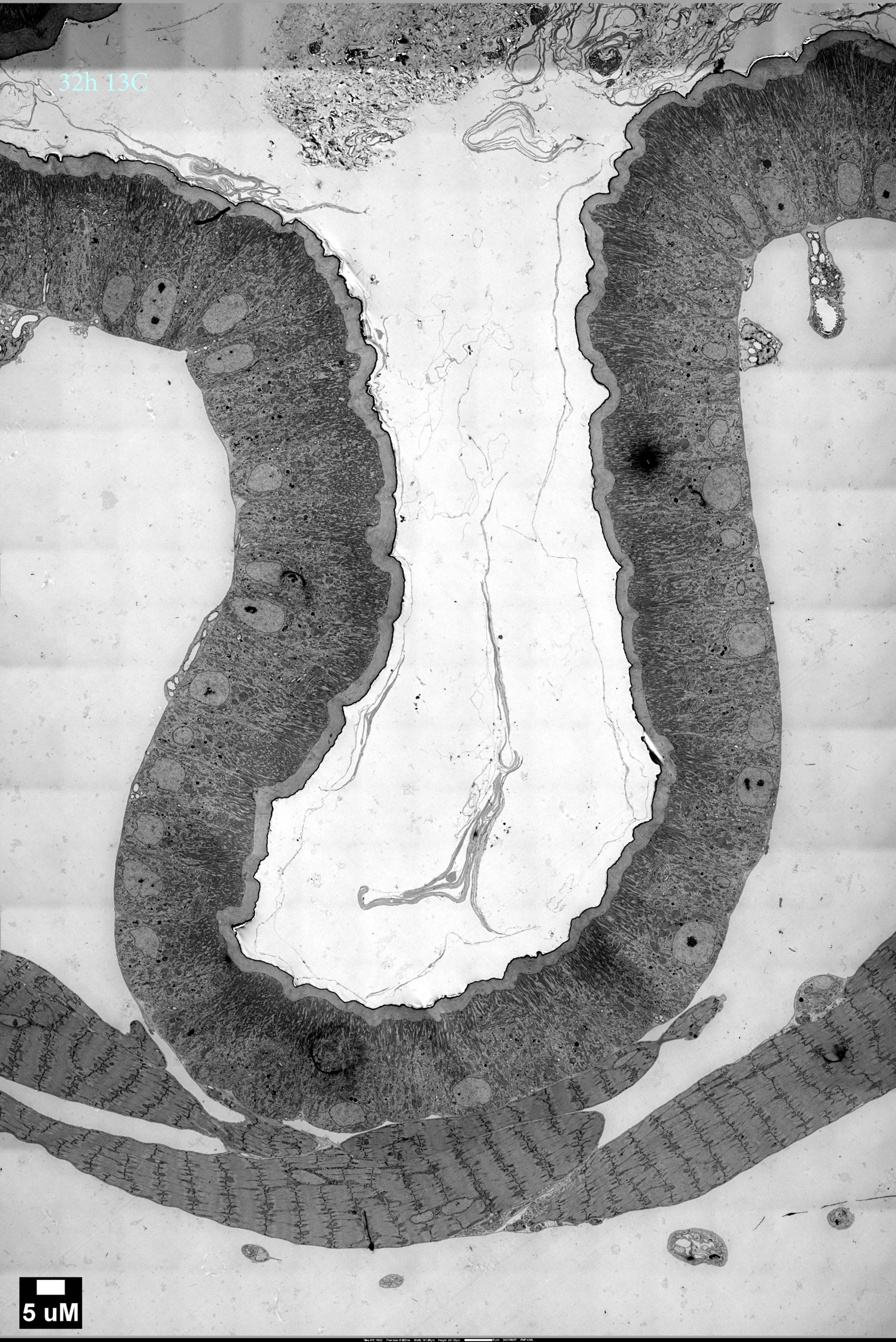

5  $\mu$ M

48h 12C

5  $\mu$ M

48h 13C

5  $\mu$ M

56h 12C

5  $\mu$ M

56h 13C

5  $\mu$ M

72h 12C

5  $\mu$ M

72h 13C

5  $\mu$ M

Supplement: Supplementary file 5 — SEM images of ileum tissue sections described in Fig. 3. [file 41564_2023_1572_MOESM5_ESM.pdf]
